# The Impact of Remote Ischemic Preconditioning on Arterial Stiffness and Heart Rate Variability in Patients with Angina Pectoris

**DOI:** 10.3390/jcm5070060

**Published:** 2016-06-23

**Authors:** Naufal Zagidullin, Elena Scherbakova, Yuliana Safina, Rustem Zulkarneev, Shamil Zagidullin

**Affiliations:** Department of Internal Diseases, Bashkir State Medical University, Ufa, 450000, Russia; alnushka555@mail.ru (E.S.); y.f.safina@mail.ru (Y.S.); zrustem@ufanet.ru (R.Z.); zshamil@inbox.ru (S.Z.)

**Keywords:** RIPC, angina pectoris, arterial stiffness, heart rate variability

## Abstract

Remote ischemic preconditioning (RIPC) is the set of ischemia episodes that protects against subsequent periods of prolonged ischemia through the cascade of adaptive responses; however, the mechanisms of RIPC are not entirely clear. Here, we aimed to study the impact of RIPC in patients with stable angina pectoris and compare it with healthy individuals with respect to arterial stiffness and heart rate variability. In the randomized, sham-controlled, crossover blind design study, a group of 30 coronary heart disease (CHD) patients (63.9 ± 1.6 years) with stable angina pectoris NYHA II-III and a control group of 20 healthy individuals (58.2 ± 2.49) were both randomly allocated for remote RIPC or sham RIPC. Arterial stiffness, pulse wave velocity (Spygmacor, Australia), and heart rate variability (HRV) were recorded before and after the procedure followed by the crossover examination. In the group of healthy individuals, RIPC showed virtually no impact on the cardiovascular parameters, while, in the CHD group, the systolic and central systolic blood pressure, central pulse pressure, and augmentation decreased, and total power of HRV improved. We conclude that ischemic preconditioning reduces not only systolic blood pressure, but also reduces central systolic blood pressure and improves arterial compliance and heart rate modulation reserve, which may be associated with the antianginal effect of preconditioning.

## 1. Introduction

More than 25 years have passed since initial research conducted by Reimer K.A. et al., (1986) [1] on the assessment in dogs of the positive effects of intermittent circumflex artery occlusion, prevented cumulative metabolic deficits and myocardial ischemic cell death. This procedure was referred to as “ischemic preconditioning” (IPC) when performed in supplying target organ arteries and remote IPC (RIPC) when provided away from the target organ, for example, on an upper or lower limb [2]. IPC has both immediate and long-term effects, the severity of which vary in different organs and have different molecular and clinical pathways [3,4]. Subsequent studies carried out in humans based on the principle of RIPC have shown that renal, cerebral, and intestinal ischemia also has a protective effect on the heart. Recently, the effect of remote and classical IP has been proven in large-scale studies on humans [5,6]. Meta-analysis in RIPC showed cardiorenal protection by reducing the incidence of myocardial infarction (both STEMI and nSTEMI) and showing less release of cardiac enzymes in patients undergoing elective percutaneous coronary interventions (PCI) [7,8,9,10]. Some larger trials are currently underway: the ERICp (The **E**ffect of **R**emote **I**schemic Preconditioning on **C**linical Outcomes) PCI trial, evaluating RIPC in STEMI patients, the EURO-CRIPS (the **EURO**pean and **C**hinese cardiac and renal **R**emote **I**schemic **P**reconditioning **S**tudy) trial, evaluating RIPC in the setting of PCI being performed for all indications apart from STEMI, and ERICCA (The **E**ffect of **R**emote **I**schemic Preconditioning on **C**linical Outcomes in Patients Undergoing **C**oronary **A**rtery Bypass Surgery), in patients with coronary artery bypass grafting. However, this effect was not present for primary endpoints in cardiovascular surgery [11], although the reduction of troponin release after coronary bypass grafting was clear [12,13].

In recent years, intensive studies on the mechanisms of RIPC in coronary heart disease (CHD) including gene and cellular levels have been conducted [14,15,16]. In patients with CHD, especially after myocardial infarction, the endothelial dysfunction is often revealed [17]. The data appeared supporting the positive impact of RIPC endothelial function based mostly on serum and tissue biomarkers [18]. Liang et al. [19] showed improvement of flow-mediated dilatation, activating a signal transducer and transcription-3 marker, and an increase in CD34+ endothelial progenitor cells found in arteries after 5 times each 4 min once daily 20 days long RIPC protocol. RIPC prevented also radial artery endothelial dysfunction induced by ischemia-reperfusion injury, probably mediated by cyclooxygenase-2 [20]. One of the leading mechanisms of RIPC in CHD seems to be neuronal signaling from the preconditioned extremity to the heart [21]. The afferent and efferent local and common neural pathways are involved in RIPC [4], because vagotomy and atropine abrogated of effects of the procedure [22,23]—so the autonomic nervous system status could be modified with RIPC, which, in turn, could be proved by the heart rate variability (HRV) test. Thus, on the one hand, the clinical efficacy of RIPC in a number of target organs was shown, and, on the other hand, the potential mechanistic pathways underlying RIPC (neural, humoral and systemic response) of IP are well investigated [24]. However, the precise clinical interactions between IP potential mechanisms and clinical RIPC efficacy are lacking. We hypothesized that RIPC could have an impact on arterial stiffness, pulse wave velocity, and HRV which might partly explain the positive impact of the procedure in coronary heart disease.

The objective was in the prospective, randomized, sham-controlled, crossover blind design study to investigate the impact of ischemic preconditioning in patients with coronary heart disease on arterial stiffness, oxygen saturation, pulse wave velocity, and heart rate variability.

## 2. Experimental Section

**Figure 1:**
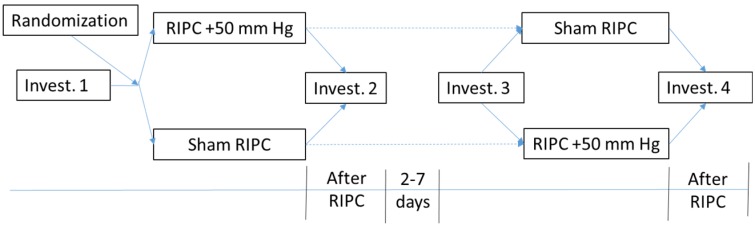
Protocol of the study both in control (*n* = 20) and CHD (*n* = 30) groups. SBP—systolic blood pressure; RIPC—ischemic preconditioning; sRIPC—sham ischemic preconditioning. The dotted arrows shows the crossover order of investigation in the groups.

In the study, 30 patients with stable angina pectoris and 20 healthy individuals in a control group were investigated. No randomization was made between the groups and the participants were recruited in the groups independently. The study design is shown in Figure 1. Patients with CHD and those from the control group were equally investigated. All the participants signed an informed consent form. The following was examined: blood pressure (BP) by Korotkov, pulse oximetry (SpO_2_), pulse wave velocity (PWV), and arterial stiffness by applanation tonometry (Sphygmacor, West Ryde, Australia). Ten-minute electrocardiography recorded in the supine position for the HRV analysis was performed before (Investigation 1) and immediately after (Investigation 2) remote RIPC. The first investigation was followed by a randomization with the help of a simple random number generator (at www.randomizer.org) for 2 groups with a probability of ½ for each choice to determine whether RIPC or sham RIPC (sRIPC) would be conducted first. RIPC was conducted by means of a three-time 5-minute’s forearm cycle cuff application with the inflation up to 50 mm Hg from the systolic blood pressure (BP) and two consecutive five-minute rests in between the cycles. Sham RIPC was conducted in a similar way, but the pressure in the cuff was pumped up to the diastolic BP (DBP) level for blinding purposes. From Day 2 to Day 7, the crossover examination (Investigation 3 and 4) was carried out, that is, the participants first applied with sRIPC underwent RIPC, while, in the second group, after RIPC the imitation was performed.

Inclusion and exclusion criteria in the CHD group.

*Inclusion criteria:*

1. CHD, stable angina NYHA II or III.
2. Signed consent form.

*Exclusion criteria:*

1. Significant arrhythmias (atrial fibrillation, atrial flutter, frequent premature atrial and ventricular beats, AV block, etc.),
2. Electric cardiostimulator,
3. Obesity, body mass index >30 kg/m^2^,
4. Peripheral arterial disease,
5. Acute coronary syndrome and myocardial infarction within 3 months,
6. Severe heart failure.

Ethical clearance for the study was approved by Ethics Committee of Bashkir state Medical University (Ufa, Russian Federation) in conformity with ethical guidelines of the 1975 Declaration of Helsinki, and all the participants gave written informed consent.

Data are presented as mean and mean error. Differences in the parameters dynamics were assessed by Student’s *t*-test for paired samples. A value of *p* < 0.05 was considered to be significant.

## 3. Results

A total of 30 patients with CHD and angina pectoris and 20 participants in the control group without CHD were investigated. Table 1 shows the basic cardiovascular characteristics of both groups. No significant changes in baseline characteristics was found between them; however, in the CHD group, the patients were older, mostly male, and shorter in height, and the arterial hypertension was more frequent—in 23 patients (76.7%) then in control group—4 patients (26.6%). In the CHD functional class NYHA II was defined in 26 patients (86.7%) and III—in 4 (13.3%).

In the control group, in accordance with randomization, 20 healthy participants were investigated in the RIPC-sRIPC (*n* = 9) or sRIPC-RIPC (*n* = 11) order, so that each one was studied four times. The summary investigation results after both examinations are shown in Table 2. The “Baseline” column presents the parameters before the test, the columns “after RIPC” and “after sRIPC” obviously present parameters after RIPC and sRIPC, and the “delta” column shows mean value differences before and after sRIPC and RIPC. No significant changes were observed before and after RIPC and sRIPC (all *p* > 0.05).

Similarly, in the CHD group, 14 patients were investigated in the RIPC–sRIPC, and 16 in the sRIPC–RIPC order. The results of the investigation of both orders are summarized in Table 3. After both sRIPC and RIPC, there was a significant decrease in the systolic BP (*p* = 0.034 in RIPC and *p* = 0.033 in sRIPC) and in the central systolic BP (*p* = 0.029 with RIPC and *p* = 0.043 in sRIPC). Only after RIPC did the Pp (*p* = 0.024) and the augmentation pulse pressure AP (*p* = 0.031) decrease significantly, whereas, in the sham group, these parameters were not significant (*p* = 0.061 and *p* = 0.089, respectively). In both groups, there was a tendency for the heart rate to decrease, the low frequency (LF) domain of HRV increased, high frequency (HF) decreased, and the ratio of LF:HF shifted in favor of HF. Among all the HRV parameters in the RIPC group, the significant increase (*p* = 0.014) in the triangular index was shown.

At the next stage, we compared the change in cardiovascular parameters after RIPC in the control group and CHD groups in order to determine the impact of RIPC on the above-mentioned parameters (Table 4). When compared with the control group, in the CHD group, the following features decreased: SBP (*p* = 0.037), central SBP (*p* = 0.019), central pulse pressure Pp (*p* = 0.016), and augmentation pressure AP (*p* = 0.048). Pulse wave velocity in the control group did not change, whereas, in the CHD group, it was insignificantly downregulated. While the HF domain of HRV tended only to decrease in the control and the CHD group, the difference between them was significant (*p* = 0.006). Furthermore, total power (TP) decreased in the control group and increased in the CHD group, which resulted in a significant difference between the two groups (*p* = 0.007).

## 4. Discussion

Ischemic preconditioning is a non-invasive effective, inexpensive method of preventing or limiting ischemic injury of the heart, lungs, and kidneys. Over the past 15 years, much research has been devoted to the mechanisms of both pre- and post-RIPC. The introduction of it into clinical practice requires extensive studies and clinical trials until RIPC becomes standard therapy. Despite a large number of studies at the molecular and clinical levels, there is still not enough data about the precise mechanisms of interaction of basic science and bedside knowledge on RIPC. RIPC effects are surely mediated by endothelium [25] by means of sympathetic and parasympathtic nervous systems and vascular and tissue agents [18,26]. The procedure effect is partly explained by the endothelium dysfunction improvement [4,19,20,21], which accompanies CHD, which in turn is associated with the rise of central blood pressure and vascular wall stiffness [27,28]. Taking into account local and general afferent and efferent sympathic and parasympathic neural pathways in RIPC, the effect on the autonomic nervous system heart rate control has also been proposed.

In our study, the impact of RIPC, compared with sham RIPC, on BP, arterial stiffness, oxygen saturation, arterial stiffness, PWV, and HRV, in both CHD patients and healthy individuals, was investigated. In the RIPC studies, the number of cuff occlusion varied from 3 to 5, and chosen protocol (3 times × 5 min), was shown to be effective in some studies [29,30,31]. As a result, both RIPC and sRIPC showed no impact on the investigated parameters in healthy participants, but, in the CHD group, RIPC improved endothelium compliance, reducing not only SBP, but also central SBP, central pulse pressure (Pp), and augmentation pressure (AP). The decrease of SBP and central SBP after sRIPC was quite a surprising finding and can be explained by venous vasodilatation, which might be caused by the pumping of the cuff in sRIPC to a diastolic level. Venous dilatation might restrict ventricle preload, which in turn may decrease the left ventricular ejection and both systolic parameters. RIPC stimulus are associated with arterial and coronary vasodilatation in animal [32] and human [33] models. According to recent studies, it has demonstrated a positive effect of RIPC on endothelial dysfunction, i.e., improving flow-mediated vasodilatation [34], increasing immunoreactivity of vasodilatative NO synthase [35], upregulating NO [36], increasing of endothelium progenitor cells [37] and vascular endothelial growth factor expression [38]. In our investigation, these pathways could explain the RIPC’s arterial vasodilation, endothelial function improvement, and positive impact on arterial stiffness (decrease in AP and Pp). Taking into account the close relation between endothelial dysfunction and coronary ischemia, the decrease in arterial stiffness after RIPC shown above may have an antianginal effect on CHD patients. RIPC seems to have an impact also on electrocardiogram—Caru M. et al., in a recent study, showed QT shortening in healthy volunteers after a RIPC protocol [39]. In our study, in CHD patients, by analyzing HRV after RIPC, the total power parameter was at about the same level, whereas the control group showed a tendency to decline, and the difference between the groups in TP was evident. Moreover, when, in the control group, the autonomic disbalance between frequency domains (LF:HF ratio) tended to increase after RIPC, in the CHD group, it did not change. RIPC could provide this effect via local afferent nerves [40,41,42] and by vagus nerve modulation [22,23]. Total power is an important marker of autonomic heart rate modulation, and upregulation may be associated with better myocardial and coronary reserve.

There are some limitations to this study. The study was a single intervention showing short-term effects that may not be sustained and therefore may not improve clinical outcomes, which demands special investigation. It is also limited by the risk of unbalanced cofounders in the basic parameters in CHD and control groups (i.e., age, gender, height, and frequency of arterial hypertension). The small number of patients may also raise the risk of random error.

## 5. Conclusions

Thus, in the randomized crossover study with RIPC and RIPC imitation in patients with CHD and angina pectoris, the decreased systolic blood pressure, arterial stiffness, and heart rate variability improvement was shown, which may be associated with the antianginal effect of the procedure. These findings may help in the understanding of the mechanisms of RIPC in CHD patients for further use in clinical practice. However, larger studies to prove this hypothesis are needed.

## Figures and Tables

**Table 1:** Characteristics of participants in coronary heart disease (CHD) and control groups.

Parameters	CHD, *n* = 30	Control, *n* = 20
Age, (in years)	63.9 ± 1.6	58.2 ± 2.49
Male (*n*)/Female (*n*)	21/9	16/4
Height, cm	169.7 ± 1.7	173.45 ± 1.5
Weight, kg	81.4 ± 2.2	82.45 ± 3.4
Body mass index, kg/m^2^	28.27 ± 1.6	27.4 ± 1.1
NYHA (*n*)		
II	26 (86.7%)	0
III	4 (13.3%)	0
Arterial hypertension, *n*	23 (76.7%)	6 (30.0%)

**Table 2:** Remote ischemic preconditioning (RIPC) and sham RIPC (sRIPC) impact on cardiovascular parameters in control group (*n* = 20).

Parameter	Sham Ischemic Preconditioning			Ischemic Preconditioning		
	Baseline	After sRIPC	Delta	Baseline	After RIPC	Delta
SBP, mm Hg	118.6 ± 2.8	115.6 ± 3.7	3.0 ± 1.8	123.1 ± 2.6	120.1 ± 2.9	3.0 ± 2.8
DBP, mm Hg	74.6 ± 1.4	75.5 ± 1.8	−0.9 ± 0.8	77.6 ± 2.2	77.1 ± 1.9	0.5 ± 2.2
Central SBP, mm Hg	108.3 ± 3.5	106.5 ± 2.5	1.8 ± 1.9	111.2 ± 2.5	110.2 ± 2.9	1.0 ± 2.9
Central DBP, mm Hg	75.9 ± 1.7	78.6 ± 1.8	−2,7 ± 2.1	78.6 ± 1.8	77.5 ± 1.8	1.1 ± 2.1
Рр, mm Hg	33.9 ± 2.1	30.6 ± 3.1	−3.3 ± 1.0	32.6 ± 2.2	32.7 ± 2.4	−0.1 ± 1.5
SpO_2_, %	97.9 ± 0.2	97.3 ± 0.28	0.6 ± 1.1	97.7 ± 0.3	97.7 ± 0.3	0.07 ± 1.4
АP (augmentation pressure), %	9.7 ± 1.8	9.0 ± 2.3	0.7 ± 1.2	6.5 ± 1.3	8.4 ± 1.5	−1.9 ± 1.4
Pp amplification, %	133.2 ± 4.5	132.7 ± 4.1	0.5 ± 3.1	140.9 ± 4.7	133.7 ± 3.8	7.2 ± 3.1
PWV, m/s	7.22 ± 0.59	7.2 ± 0.49	−0.02 ± 0.19	7.33 ± 0.63	7.35 ± 0.6	−0.02 ± 0.29
Heart rate, min	68.1 ± 2.8	65.5 ± 2.7	2.6 ± 0.77	68.1 ± 2.8	65.5 ± 2.7	2.6 ± 0.87
Triangular index	6.85 ± 0.51	7.1 ± 0.51	−0.25 ± 0.5	5.75 ± 0.28	6.4 ± 0.6	−0.65 ± 0.6
LF norm	59.4 ± 5.1	66.6 ± 4.4	−7.2 ± 4.2	58.9 ± 8.5	68.9 ± 8.4	−10.0 ± 11.1
HF norm	40.6 ± 5.1	33.4 ± 4.4	7.2 ± 4.5	41.1 ± 8.5	31.0 ± 4.8	10.1 ± 10.5
LF:HF ratio	2.0 ± 0.39	3.0 ± 0.74	−1.0 ± 0.7	1.43 ± 0.9	2.22 ± 0.9	−0.79 ± 1.0
TP	753.1 ± 300.2	661.9 ± 151.6	91.2 ± 91.1	1096.3 ± 431.5	627.1 ± 238.5	469.2 ± 91.1

P.s.: *p* < 0.05 comparison (delta) of the parameter before and after RIPC/sRIPC.

**Table 3:** Changes in cardiovascular parameters after sRIPC/RIPC in CHD group (*n* = 30).

Parameter	Sham Ischemic Preconditioning			Ischemic Preconditioning		
	Base	After sRIPC	∆	Base	After RIPC	∆
SBP, mm Hg	137.0 ± 3.3	120.1 ± 3.2 *	16.9 ± 2.4	134.7 ± 5.3	119.4 ± 4.0 *	15.3 ± 3.5
DBP, mm Hg	84.7 ± 1.8	77.7 ± 1.8	7.0 ± 1.4	79.2 ± 2.7	75.2 ± 2.3	4.0 ± 2.8
Central SBP, mm Hg	134.9 ± 3.7	122.1 ± 3.8 *	12.8 ± 3.1	132.9 ± 5.8	118.5 ± 4.0 *	14.4 ± 3.9
Central DBP, mm Hg	84.0 ± 1.9	78.4 ± 2.0	5.6 ± 1.6	78.7 ± 2.8	76.1 ± 2.3	2.6 ± 1.8
Pp, mm Hg	50.7 ± 2.8	44.1 ± 2.9	6.6 ± 2.1	54.3 ± 4.2	42.25 ± 2.3 *	12.0 ± 3.9
SpO_2_, %	97.2 ± 0.13	97.93 ± 0.2	−0.73 ± 0.25	97.2 ± 0.4	97.3 ± 0.3	−0.1 ± 0.3
АP, %	127.2 ± 1.8	125.8 ± 2.7	1.4 ± 2.05	131.4 ± 2.6	127.2 ± 1.8 *	4.2 ± 2.1
Pp, amplification, %	12.3 ± 1	11.68 ± 1.2	0.62 ± 1.39	12.3 ± 1.0	11.7 ± 1.2	0.5 ± 1.5
PWV, m/sec	5.5 ± 0.6	6.0 ± 0.63	−0.5 ± 0.3	5.37 ± 0.8	4.95 ± 0.49	0.42 ± 0.53
Heart rate, min	62.5 ± 2.2	61.2 ± 2.4	1.3 ± 0.9	68.6 ± 3.1	63.9 ± 3.0	4.7 ± 1.16
Triangular index	5.78 ± 0.46	6.7 ± 0.59	−0.92 ± 0.7	6.0 ± 0.46	9.0 ± 1.1 *	−3.0 ± 1.04
LF norm	26.4 ± 3.6	31.8 ± 4.2	−5.4 ± 3.8	42.7 ± 5.5	48.1 ± 7.2	−5.4 ± 2.2
HF norm	73.6 ± 3.6	68.2 ± 4.2	5.4 ± 3.8	57.3 ± 5.5	55.5 ± 7.2	1.8 ± 4.27
LF:HF ratio	0.38 ± 0.07	0.51 ± 0.12	−0.13 ± 0.11	0.75 ± 0.3	0.87 ± 0.25	−0.12 ± 0.2
TP	2767.5 ± 1912.6	1736.1 ± 544.9	1031.4 ± 747.7	1077.4 ± 451.5	1156.1 ± 223.5	−78.7 ± 84.2

P.s.: * *p* < 0.05 comparison (delta) of parameter before and after RIPC/sRIPC.

**Table 4:** Comparison of changes in the parameters in RIPC in the control and CHD groups.

Parameters	∆ RIPC, Control Group, *n* = 20	∆ RIPC, CHD Group, *n* = 30	*p*
SBP, mm Hg	3.0 ± 2.8	15.3 ± 3.5 *	0.028
DBP, mm Hg	0.5 ± 2.2	4.0 ± 2.8	0.19
Central SBP, mm Hg	1.0 ± 2.9	14.4 ± 3.9 *	0.009
Central DBP, mm Hg	1.1 ± 2.1	2.6 ± 1.8	0.31
Pp, mm Hg	−0.1 ± 1.5	12.0 ± 3.9 **	0.008
SpO_2_, %	0.07 ± 1.4	−0.1 ± 0.3	0.26
АP, %	−1.9 ± 1.4	4.2 ± 2.1 *	0.041
Pp, amplification,%	7.2 ± 3.1	0.5 ± 1.5	0.086
PWV, m/s	−0.02 ± 0.29	0.42 ± 0.53	0.101
HR, beat/min	2.6 ± 0.87	4.7 ± 1.16	0.066
Triangular index	−0.65 ± 0.6	−3.0 ± 1.04	0.057
LF norm	−10.0 ± 11.1	−5.4 ± 2.2	0.47
HF norm	10.1 ± 10.5	1.8 ± 4.27	0.067
LF:HF ratio	−0.79 ± 1.0	−0.12 ± 0.2	0.076
TP	469.2 ± 91.1	−78.7 ± 84.2 **	0.006

P.s.: * *p* < 0.05 and ** *p* < 0.05 comparison (delta) of parameter before and after RIPC/sRIPC.

## Abbreviations

The following abbreviations are used in this manuscript:

AP: pulse augmentation
CHD: coronary heart disease
DBP: diastolic blood pressure
HF: high frequency low frequency (domain)
HR: heart rate
HRV: heart rate variability
RIPC: ischemic preconditioning
LF: low frequency low frequency (domain)
NYHA: New York Heart Association
PCI: percutaneous coronary interventions
Pp: pulse pressure
PWV: pulse wave velocity
SBP: systolic blood pressure
sRIPC: sham ischemic preconditioning
S_P_o2: oxygen saturation
STEMI: ST-elevated myocardial infarction
nSTEMI: non ST-elevated myocardial infarction
TP: total power
